# Use of patient-derived xenograft mouse models in cancer research and treatment

**DOI:** 10.4155/fsoa-2017-0136

**Published:** 2017-12-07

**Authors:** Erica Yada, Satoshi Wada, Shintaro Yoshida, Tetsuro Sasada

**Affiliations:** 1 Division of Cancer Immunotherapy, Kanagawa Cancer Center Research Institute, Yokohama, Japan; 2 Cancer Vaccine Center, Kanagawa Cancer Center, Yokohama, Japan

**Keywords:** biobank, biomarker discovery, cancer, drug screening, mouse model, patient-derived xenograft, personalized medicine, preclinical research

## Background of patient-derived xenograft mouse models

Since most advanced cancers are still incurable, basic, preclinical and clinical cancer research remains necessary for developing new therapeutic modalities. Many cancer cell lines have been developed, which have for a long time been available for use in basic and preclinical cancer research. However, those cell lines have the disadvantage that they do not necessarily reflect the behaviors of the original cancer cells in patients, owing to the artificial nature of their culture conditions. Therefore, cell line-derived xenograft tumor models, which are established by transplanting well-validated cancer cell lines into immunocompromised mice, have also been used for cancer research [1]. Cell line-derived xenograft has the advantage of creating microenvironments closer to the tumor's physiological and pathological conditions, but also has the disadvantage that the cancer cells employed might have already lost some of their original characteristics through adaptations to *in vitro* growth.

Patient-derived xenograft (PDX) mouse models have attracted attention in recent years, with the aim of resolving such problems. PDX mouse models are established by direct engraftment of patient-derived tumor fragments into immunocompromised mice. Since PDXs have been suggested to retain morphologies, architectures and molecular signatures very close to those of the original tumors, it is probable that they have great potential for both basic and preclinical cancer research [2], such as biomarker discovery, drug screening for personalized medicine, understanding of drug-resistance mechanisms and novel therapy development.

## Characteristics of PDX models

There have been several experimental protocols reported to generate PDX models, as individual research groups have their own ways to improve the success rate of PDX engraftment, although the protocols seem to share the fundamental concepts and techniques. Briefly, pieces of solid tumors or single-cell suspensions are collected from tumor tissues obtained by surgery or biopsy, and are transplanted under the skin (subcutaneous transplantation), in the same organ as the original tumors in the patients (orthotopic transplantation), or in the renal capsule in the recipient immunocompromised mouse. Subcutaneous transplantation models allow for easier cell transfer and precise monitoring of tumor formation and growth [3]. In contrast, orthotopic PDX models are more difficult than heterotopic subcutaneous models for transplantation techniques and monitoring of tumor growth, but the microenvironments of transplanted tumors might be more similar to those of the original tumors in the patients. For example, it was reported that orthotopic PDX models showed increased incidence of metastases from transplanted pancreatic tumors, compared with heterotopic subcutaneous models [4].

There has been a lot of discussion regarding whether tumor cells in PDX models show characteristics similar to those of the original tumors. For example, it was reported that although human breast cancer cell lines were often poorly metastatic, the majority of breast cancer PDXs showed metastases as seen in the original cancers [5]. Regarding morphological aspects, it was shown that cellular and structural characteristics were well maintained in the PDXs from various kinds of cancers [6]. Moreover, most PDXs were reported to preserve genomic alterations and global gene expression profiles, compared with those from the original cancers [6,7]. Notably, however, it was recently suggested that PDXs display some genomic clonal selection and might be more genomically unstable than previously thought [8]. For example, Ben-David *et al*. analyzed the dynamics of DNA copy number alterations during PDX passaging across 24 types of cancer [9]. Despite overall similarity, the copy number alteration landscapes of PDX models gradually shifted away from those of the original primary tumors, although such selection pressure was not well understood.

PDXs seem to be a valuable model for cancer research, although it may be important to know their limitations as well. In original tumor tissues, stromal cells such as epithelial cells and fibroblasts co-exist with cancer cells, whereas in PDX models, almost all stromal cells derived from human tumors cannot proliferate continuously and are replaced by cells derived from the recipient mouse. Therefore, there are unavoidable limitations to studying tumor microenvironments using PDX models. In addition, the immune system is compromised in the mice employed for PDX models, such as nude mice (T cell-deficient), severe combined immunodeficient mice (T- and B cell-deficient) and extremely immunodeficient mice [T-, B-, and NK cell-deficient; NOG mice (NOD.Cg-*Prkdc^scid^Il2rg^tm1Sug^*/ShiJic) and NSG mice (NOD.Cg-*Prkdc^scid^Il2rg^tm1Wjl^*/SzJ)]. Indeed, the effects of cancer immunotherapy, using agents such as immune checkpoint inhibitors, might be difficult to evaluate with PDXs transplanted into these immune cell-deficient mice [10]. Establishing PDX models in more immunocompetent mice might be essential to investigate cancer immunotherapies.

## Application of PDX models for basic & preclinical cancer research

One of the most useful applications of PDX models in basic research might be to clarify therapeutic mechanisms, as well as to identify targets or biomarkers for cancers. For example, Das Thakur *et al*. demonstrated that cells resistant to the BRAF inhibitor vemurafenib also showed drug dependency by using two melanoma PDX models, in which resistant cells were selected by continuous vemurafenib treatment [11]. This finding suggested a potential therapeutic strategy to prevent the emergence of lethal drug resistance by altered dosing in melanoma patients with BRAF mutations [11]. In addition, Zhao *et al*. screened for the expression of cancer stem cell markers through qPCR analysis and reported that high consistency in the prognostic value of the expression of CD133/CD44 was observed in both hepatocellular carcinoma patients and the PDX models [12]. These applications of preclinical PDX models might be valuable, as they allow us to save time and costs required for clinical evaluations.

Another useful application of PDX models might be to make treatment decisions for personalized medicine by screening drugs in preclinical models. Although cancer cells isolated from tumor tissues have been directly used for anticancer drug screening, they showed limited value in accurately predicting clinical response. However, PDX models could be used as more reliable ‘avatars’ in drug screening for personalized cancer treatment. For example, Hidalgo *et al*. established pancreatic PDX models from 14 patients, and screened 63 anticancer drugs in 232 treatment regimens. Following identification of the most effective treatment regimens in the PDX models, the 17 regimens were tried in 11 patients, of whom durable partial remission was detected in 15 treatments [13]. This strategy of screening drugs seems to be very effective and promising, although it may sometimes have limitations. In fact, it usually takes several months (4–30 weeks with an average time of 14 weeks) to establish PDX models, potentially dependent on the recipient mouse strains, tumor types or percentages of cancerous cells within the patient tissues resected for engraftment [3]. In addition, the success rate of establishment of PDX is also reported to be limited from 23 to 75% [14]. Therefore, there persist several hurdles to the use of ‘avatars’ for personalized screening of appropriate drugs for individual patients, despite ongoing clinical trials.

## Future perspective

PDXs can be stored in frozen conditions as a tumor biobank, to be available for re-transplantation and expansion as soon as they are required for experiments [15]. In addition, even if the sizes of original tumors derived from patients are small, the tumors engrafted as PDXs can be continuously expanded to larger volumes in immunocompromised mice. Large and diverse collections of PDX models thus allow us to efficiently and precisely test anticancer drugs [16]. Indeed, some drugs can be screened at once by using many different PDX models that might retain their idiosyncratic characteristics of different tumors from different patients. Therefore, PDX biobanks could represent a powerful resource for preclinical cancer pharmacogenomic studies.

Recently, a network of PDX banking of many research collaborations with potential success has been established [17]. For example, EurOPDX is a scientific network of non-for-profit research institutions, mainly in Europe (http://europdx.eu/) [17]. They share over 1500 PDX models from more than 30 different solid tumor types, as well as information on their characteristics. For example, Bruna *et al*. well-characterized the breast cancer PDXs in this biobank, and also prepared PDX-derived tumor cells for culture, which preserved the characteristics of the original PDXs. They developed a platform of high-throughput drug screening assays with PDX-derived tumor cells, on which drug responses can be assessed and validated [18]. Considering such useful applications of a network of PDX banking, more attention should be paid to PDX models for basic and preclinical research, such as biomarker discovery, drug screening for personalized medicine, understanding of drug resistance mechanism and novel therapy development.

## References

[1] Sausville EA, Burger AM (2006). Contributions of human tumor xenografts to anticancer drug development. *Cancer Res.*.

[2] Cho SY, Kang W, Han JY (2016). An integrative approach to precision cancer medicine using patient-derived xenografts. *Mol. Cells*.

[3] Kim MP, Evans DB, Wang H (2009). Generation of orthotopic and heterotopic human pancreatic cancer xenografts in immunodeficient mice. *Nat. Protoc.*.

[4] Fu X, Guadagni F, Hoffman RM (1992). A metastatic nude-mouse model of human pancreatic cancer constructed orthotopically with histologically intact patient specimens. *Proc. Natl Acad. Sci. USA*.

[5] DeRose YS, Wang G, Lin YC (2011). Tumor grafts derived from women with breast cancer authentically reflect tumor pathology, growth, metastasis and disease outcomes. *Nat. Med.*.

[6] Chijiwa T, Kawai K, Noguchi A (2015). Establishment of patient-derived cancer xenografts in immunodeficient NOG mice. *Int. J. Oncol.*.

[7] Gao H, Korn JM, Ferretti S (2015). High-throughput screening using patient-derived tumor xenografts to predict clinical trial drug response. *Nat. Med.*.

[8] Eirew P, Steif A, Khattra J (2015). Dynamics of genomic clones in breast cancer patient xenografts at single-cell resolution. *Nature*.

[9] Ben-David U, Ha G, Tseng YY (2017). Patient-derived xenografts undergo mouse-specific tumor evolution. *Nat. Genet.*.

[10] Hidalgo M, Bruckheimer E, Rajeshkumar NV (2011). A pilot clinical study of treatment guided by personalized tumorgrafts in patients with advanced cancer. *Mol. Cancer Ther.*.

[11] Das Thakur M, Salangsang F, Landman AS (2013). Modelling vemurafenib resistance in melanoma reveals a strategy to forestall drug resistance. *Nature*.

[12] Zhao Q, Zhou H, Liu Q (2016). Prognostic value of the expression of cancer stem cell-related markers CD133 and CD44 in hepatocellular carcinoma: from patients to patient-derived tumor xenograft models. *Oncotarget*.

[13] Hidalgo M, Amant F, Biankin AV (2014). Patient-derived xenograft models: an emerging platform for translational cancer research. *Cancer Discov.*.

[14] Lai Y, Wei X, Lin S (2017). Current status and perspectives of patient-derived xenograft models in cancer research. *J. Hematol. Oncol.*.

[15] Alkema NG, Tomar T, Duiker EW (2015). Biobanking of patient and patient-derived xenograft ovarian tumour tissue: efficient preservation with low and high fetal calf serum based methods. *Sci. Rep.*.

[16] Hidalgo M, Kung AL, Aparicio S (2015). Examining the utility of patient-derived xenograft mouse models. *Nat. Rev. Cancer*.

[17] Byrne AT, Alférez DG, Amant F (2017). Interrogating open issues in cancer medicine with patient-derived xenografts. *Nat. Rev. Cancer*.

[18] Bruna A, Rueda OM, Greenwood W (2016). A biobank of breast cancer explants with preserved intra-tumor heterogeneity to screen anticancer compounds. *Cell*.

